# Dual-variable control load-sensing system for multi-way valves in hilly and mountainous tractors

**DOI:** 10.1371/journal.pone.0321940

**Published:** 2025-05-08

**Authors:** Yiwei Wu, Yuzhen Ruan, Jingyun Zhang, Shuai Wang, Lin Wang, Xiaoxiao Du, Xianghai Yan

**Affiliations:** 1 Henan University of Science and Technology, College of Vehicle and Traffic Engineering, Luoyang, China; 2 State Key Laboratory of Intelligent Agricultural Power Equipment, Luoyang, China; 3 Henan University of Science and Technology, School of Mechatronics Engineering, Luoyang, China; 4 Luoyang Tractor Research Institute Co., Ltd., Luoyang, China; 5 YTO Group Corporation, Luoyang, China; ICAR-IISWC: ICAR Indian Institute of Soil and Water Conservation, INDIA

## Abstract

To tackle the challenges of severe pressure loss, mismatched flow supply and demand, and high flow demands at pump outlets commonly seen in multi-way valves of hilly and mountainous tractors, a load-independent flow distribution system was developed. This system combines an electro-hydraulic load-sensing pump with an independent flow distribution mechanism at the load port, resulting in a dual-variable load sensing system that integrates variable displacement and variable speed control. Using AMESim software, the core components and system model of the dual-variable load sensing system were built. Considering the operational characteristics of the multi-way valve, a load-sensitive variable pump with pressure differential and displacement detection was employed as the feedback control unit. The system incorporates a valve post-compensation mechanism and a three-position, six-way electro-hydraulic proportional directional valve. Additionally, a self-adjusting motor control method for pressure differential adaptation was introduced to regulate system pressure under high-load conditions. Simulation analyses of the system performance revealed that the design effectively boosts energy efficiency, achieving average energy savings of 20% and reaching up to 50% under optimal conditions, while keeping pressure fluctuations at the valve port within a 5% margin. By employing dual-variable load sensing technology, this system substantially improves the energy efficiency and operational effectiveness of multi-way valves while maintaining stable pressure throughout the operation. The design offers a reliable technical framework for the development and optimization of multi-way valves in hilly and mountainous tractors.

## Introduction

Hilly and mountainous regions play a critical role in agricultural production. The complex terrain and diverse operational demands in these areas impose higher requirements on agricultural machinery [1–3]. To address the unique challenges posed by such terrain, it is essential to develop specialized tractors for hilly and mountainous regions, aiming to enhance the level of agricultural mechanization [4–7]. Among these, the multi-way valve system, as a core component of the tractor’s hydraulic system, is primarily responsible for controlling key hydraulic parameters such as flow, pressure, and direction to achieve various operational functions [8–11]. Given that the operational requirements in hilly and mountainous areas are significantly more demanding compared to plains, there is an urgent need to develop a multi-way valve system with real-time independent flow distribution capabilities to meet the specific demands of these challenging environments [12, 13].

Currently, significant progress has been made by domestic and international scholars in the research of multi-way valve systems. These studies have not only driven technological advancements in multi-way valve systems but also provided critical support for the development of agricultural machinery in hilly and mountainous regions. As agricultural modernization continues to advance in these areas, enhancing the performance and adaptability of multi-way valve systems in complex terrains is crucial to meeting diverse agricultural operation demands.Lei Ge et al. [14] proposed a pump/valve combined control strategy that integrates pump speed and displacement control with an independent metering system to improve the energy efficiency and dynamic response of hydraulic excavators. The results demonstrated that, compared to fixed-speed strategies, this method significantly reduced idle and partial-load energy consumption and improved energy efficiency by 3%–10%. Qiankun Li et al. [15] introduced a flow-matching method with pump-valve synchronous control. By adjusting the speed of asynchronous motors and controlling the flow area of the main valve port in the multi-way valve, they matched the load-required flow with the pump output flow in the LUDV hydraulic system of electric excavators, effectively reducing overflow losses and improving energy-saving performance. Tianliang Lin et al. [16] developed a load-sensing system based on displacement adaptation and variable-speed control to address flow matching issues under varying load conditions in electric excavators. Their results showed that the system effectively enhanced control performance and reduced energy consumption by 19%–70%.Bury P et al. [17] analyzed the start-up process of hydrostatic transmission system under proportional valve control, focusing on dynamic residual pressure, energy consumption and speed characteristics. The influence of PI control system on pressure, speed and start-up time is studied by establishing a mathematical model with concentrated parameters and combining MATLAB-Simulink simulation and experimental verification. The improved proportional valve polynomial model improves the simulation accuracy, and experiments show that adjusting the shape of the control signal (e.g., ramp time) can reduce the dynamic pressure peak (e.g., from 9.29 MPa to 3.53 MPa) and optimize the energy efficiency. The closed-loop control further reduces the steady-state error and overshoot, and verifies the effectiveness of the model in optimizing the dynamic performance and reliability of the system.Ali Volkan Akkaya [18] proposed a hybrid fuzzy-PID control strategy to solve precise positioning challenges in hydraulic systems, demonstrating that this strategy outperformed other controllers, offering shorter rise and settling times while minimizing the integral absolute error and integral time absolute error.Gao et al. [19] introduced a novel dual-actuator hydraulic system to address the high energy consumption and low energy efficiency of traditional hydraulic systems. Their findings showed that the system reduced energy consumption by 17.34% and increased productivity by 18.85%.Karpenko, M et al[20] studied the effects of the connection of pipe to corner fitting (45° and 90°) on fluid flow characteristics, energy loss and operating delay in an aircraft hydraulic drive system. A CFD numerical model based on Navier-Stokes equations and k-epsilon turbulence model was established to analyze the pressure drop, eddy current formation and energy loss of the fluid in the pipeline, and compared with the traditional equivalent length method (EL and EL-SS). The study found that the pressure loss of the actual corner fitting was 15–26% higher than that of the conventional method, and the operating delay was significantly increased (about 13% at 45° and 33% at 90°). The simulation method of grid independence verification reveals the shortcomings of the traditional equivalent length method in the design of complex corner pipe fitting, and provides theoretical support for the optimization of hydraulic system design.Amirante et al. [21] investigated the driving force of the spool valve in an open-center hydraulic directional control valve using experimental and numerical methods. They found that during the initial opening phase, flow forces facilitated spool opening, but at later stages, these forces impeded it as they reached their maximum value.Ivan Okhotnikov et al. [22] presented a new design for a rotary hydraulic flow control valve. Through computational fluid dynamics (CFD) analysis, they addressed flow control and pressure loss issues in high-flow hydraulic systems, concluding that the design effectively reduced flow-induced forces and improved control precision and reliability. Paloniitty et al. [23] proposed a new digital hydraulic valve system that uses multiple equally sized switching valves with cyclic switching control algorithms. This design improved the resolution and linearity of digital hydraulic valves, achieving compact, robust, and high-performance valve control.Yongkui Fan et al. [24] designed a hydraulic multi-point power output system tailored for complex operations in hilly and mountainous tractors. This system resolved the challenge of traditional systems being unable to meet simultaneous multi-channel hydraulic output demands. Their results demonstrated that the system could achieve functions such as load feedback, pressure compensation, and flow distribution, effectively meeting the operational requirements of hilly and mountainous tractors.

This study addresses the shortcomings of existing tractor multi-way valve systems, such as flow saturation caused by load fluctuations, poor low-speed maneuverability, and low energy efficiency. A dual-variable load-sensing control system based on pressure differential and displacement adaptive variable-speed control is proposed. The system adjusts the flow rate of the variable pump based on load demands, effectively avoiding unnecessary idling and throttling losses. Through PID control, the variable-speed motor expands the flow matching range of the power transmission system, ensuring that the displacement-adaptive variable-speed power source operates in a high-efficiency range.An AMESim simulation model was built to verify the effectiveness of the proposed system. The results demonstrate that this multi-way valve system provides a high-precision, low-energy-consumption solution for hilly and mountainous tractors, establishing a solid foundation for their application in complex terrains.

## A novel dual-variable load-sensing system based on load sensing and variable speed control

**Fig 1:**
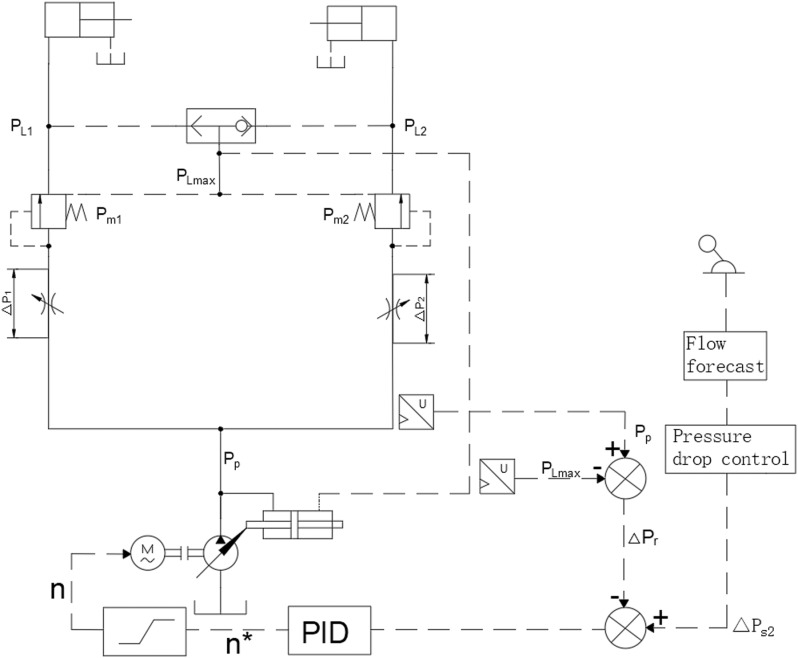
Simplified diagram of a multi-way valve principle.

The new dual-variable control load sensing system based on load sensing and variable speed control is shown in Fig 1. Variable speed control of the variable motor is used to drive the load-sensitive variable pump and provide power for the dual-variable control load sensing system. When the system’s traffic requirements change, the controller combines the value of the variable pump outlet pressure and the load sensitive valve pressure after differential calculation with the flow prediction value, and outputs the motor speed signal after multi-variable decoupling control through PID, so that the system can realize stepless speed regulation within the maximum displacement range. The load sensitive valve is adjusted by the pressure of the shuttle valve feedback oil line LS, and determines the pressure difference of the differential pressure displacement detection device in parallel with the load sensitive valve. The differential pressure displacement detection device transfers the pressure difference of the valve core to the variable pump, and the motor speed is affected by the feedback oil line pressure of the load sensitive valve. In theory, when the independent flow distribution system works, the output pressure of the variable pump displacement and the variable motor speed will reach the required flow and pressure of the hydraulic cylinder with the minimum power and the fastest speed[25].

**Fig 2:**
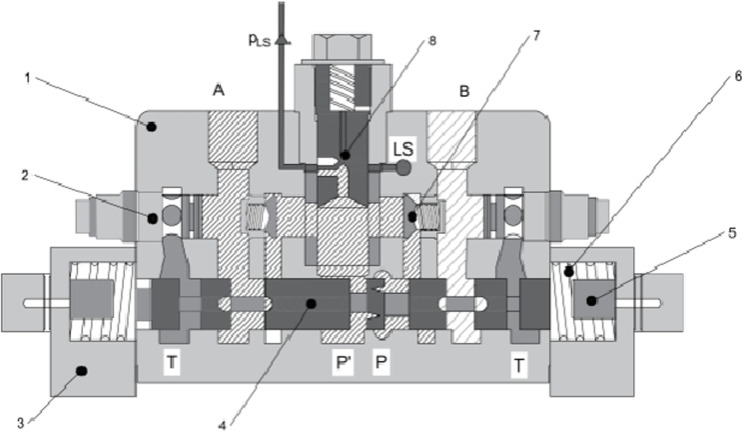
LUDV Multi-way Valve Structure Principle 1. Valve body 2.Secondary safety valve 3.End cover 4.Directional control valve core5.Stroke limit 6.Reset spring 7.Check valve 8.Pressure compensator.

The structural principle of the load-independent flow distribution (LUDV) multi-way valve is shown in Fig 2. The key difference between the LUDV system and the load-sensing (LS) system lies in the sequence of throttling and pressure compensation. In the LUDV system, pressure compensation occurs after throttling, while the LS system performs the opposite. In the LUDV system, the pressure compensation valve is placed after the variable throttle port of the directional control valve. Since tractor directional control valves are typically double-acting with two oil circuits, A and B, to avoid adding a pressure compensation valve to both circuits and thereby reducing structural complexity, the directional control valve’s reversing section is located after the pressure compensation valve [26].In a hydraulic system, when multiple actuators perform composite operations simultaneously, flow is often preferentially supplied to the actuator with the lighter load. This can cause interference among the composite operations, leading to a “flow competition” phenomenon. The LUDV multi-way valve uses the highest load-sensing pressure as feedback pressure for pressure compensation after the main valve throttle port. This ensures that the pressure difference before and after each branch throttle valve is equal, and the flow to each actuator is proportional only to the flow area of the respective main valve port, regardless of the load pressure [27].

As an example of the directional control valve moving to the right, when the outlet is at port A, the hydraulic oil from the hydraulic pump outlet enters the P chamber of the directional control valve. It flows through the throttling groove on the main valve spool of the directional control valve and enters the P’ chamber. The pressurized oil in the P’ chamber passes through the pressure compensator (8). At this point, the oil splits into two paths: one flows into the Ls port, acts on the shuttle valve, and then on the load-sensing valve; the other enters the check valve on the A-port side. After the check valve is opened, the oil enters the oil channel on the A-port side. Since the valve spool has moved to the right, the A-port side valve opens, and the oil flows through the directional control valve into port A [28, 29].

## Performance analysis of the dual-variable load system

The system adopts a dual-variable control method using a variable pump and a variable motor. For simplification during calculations, the mathematical model neglects the effects of safety valves, check valves, and pipelines. Assuming that hydraulic oil is an ideal Newtonian fluid, the compressibility of hydraulic oil and the change of oil viscosity with temperature are ignored, and the fluid density remains constant, and the turbulence effect is ignored. It is assumed that the system has no internal and external leakage and maintains a completely sealed state, and the pipeline system is regarded as a rigid connection, ignoring its cavity effect and pressure wave transmission. It is considered that each control loop is completely decoupled without cross-coupling interference. Set the mechanical transmission efficiency to 100%, ignore the mechanical friction loss of the pump-motor assembly, and ignore the impact of the dead zone on the overall hydraulic system. Based on the above simplified model, the system’s performance is analyzed.

As shown in Fig 1, the transmission model of the motor and pump can be expressed as:


$${\text{T}}_{\text{em}}-\frac{{\text{P}}_{\text{p}}\text{V}}{2\text{π}}=\text{J}\frac{{\text{dw}}_{\text{p}}}{\text{dt}}$$ (1)

Where, T_em_—Motor torque, Nm;

P _p_—Pump outlet pressure, MPa;

V —Pump outlet displacement, cc;

J —Equivalent rotational inertia of the powertrain, kg·m^2^；

w_p_—Pump angular velocity, rad/s.

The motor’s output torque is determined by the load pressure, with the formula as follows.


$${\text{T}}_{\text{em}}=\frac{\text{V}}{2\text{π}}{\text{p}}_{\text{L}}\left(\text{s}\right)$$ (2)

Where, p_L_—load pressure, MPa.

The dynamic model of flow pressure out of the mouth can be written as:


$${\text{Q}}_{\text{p}}-\frac{{\text{w}}_{\text{p}}\text{V}}{2\text{π}}=\frac{{\text{V}}_{\text{p}}}{\text{β}}\frac{{\text{dp}}_{\text{p}}}{\text{dt}}+{\text{C}}_{1}{\text{p}}_{\text{p}}$$ (3)

Where, Q_p_—pump flow, L/min;

β—bulk modulus of oil, MPa;

C_1_—Leakage factor, Pa/min.

The load-sensing pump throttle valve and compensating relief valve can form a flow control valve. The flow equation for the load-sensing valve can be written as follows:


$${\text{Q}}_{\text{p}}={\text{K}}_{\text{x}}{\text{x}}_{\text{v}}+{\text{K}}_{\text{p}}\left({\text{p}}_{\text{p}}-{\text{p}}_{\text{L}}\right)$$ (4)

Where, K_x_—flow gain, (L/min)/m;

K_p_—flow pressure coefficient, (L/min)/MPa.

The transfer function of load sensitive valve spool displacement can be expressed as:


$${\text{x}}_{\text{v}}\left(\text{s}\right)=\frac{{\text{x}}_{\text{g}}}{{\text{τ}}_{1}\text{s}+1}$$ (5)

Where, τ_1_—response time constant, s.

The flow pressure equation of the spool oil can be expressed as:


$${\text{Av}}_{\text{c}}-{\text{Q}}_{\text{c}}=\frac{{\text{V}}_{\text{c}}}{\text{β}}\frac{{\text{dp}}_{\text{L}}}{\text{dt}}+{\text{C}}_{1}{\text{p}}_{\text{L}}$$ (6)

Where, A—piston area of the cylinder, m^2^;

V_c_—piston movement speed of the cylinder, m/s.

The mechanical transmission model of the hydraulic cylinder can be given as:


$${\text{p}}_{\text{L}}\text{A}-{\text{F}}_{\text{L}}={\text{m}}_{\text{L}}\frac{{\text{dv}}_{\text{c}}}{\text{dt}}$$ (7)

Where, F_L_—load force, N;

m_L_—load mass, kg.

Based on the above equation, the flow transfer function of the load-sensitive valve can be represented as:


$${\text{Q}}_{\text{p}}\left(\text{s}\right)={\text{K}}_{\text{x}}{\text{G}}_{\text{x}}\left(\text{s}\right){\text{x}}_{\text{v}}\left(\text{s}\right)+{\text{K}}_{\text{p}}{\text{G}}_{\text{p}}\left(\text{s}\right){\text{p}}_{\text{L}}\left(\text{s}\right)$$ (8)

Where,


$${\text{G}}_{\text{x}}=\frac{\frac{{\text{V}}_{\text{p}}}{\text{β}}4{\text{π}}^{2}{\text{Js}}^{2}+4{\text{πJC}}_{1}\text{s}+{\text{V}}^{2}}{\frac{{\text{V}}_{\text{p}}}{\text{β}}4{\text{π}}^{2}{\text{Js}}^{2}+\left(4{\text{π}}^{2}{\text{JC}}_{1}+{\text{K}}_{\text{x}}4{\text{π}}^{2}\text{J}\right)\text{s}+{\text{V}}^{2}}$$ (9)

$${\text{G}}_{\text{p}}=\frac{\frac{{\text{V}}_{\text{p}}}{\text{β}}4{\text{π}}^{2}{\text{Js}}^{2}+4{\text{πJC}}_{1}\text{s}}{\frac{{\text{V}}_{\text{p}}}{\text{β}}4{\text{π}}^{2}{\text{Js}}^{2}+\left(4{\text{π}}^{2}{\text{JC}}_{1}+{\text{K}}_{\text{p}}4{\text{π}}^{2}\text{J}\right)\text{s}+{\text{V}}^{2}}$$ (10)

K_x_G_x_(s) and K_p_G_p_(s) represent the dynamic relationships between the pump flow rate and displacement, and the opening area of the load-sensing valve in the system, respectively.Then, the natural frequency and damping ratio of the system can be expressed as:


$$\text{ω}=\sqrt{\frac{{\text{A}}^{2}\text{β}}{{\text{V}}_{\text{c}}{\text{m}}_{\text{L}}}}$$ (11)

$$\text{ξ}=\frac{{\text{K}}_{\text{p}}{\text{G}}_{\text{p}}\left(\text{s}\right)+{\text{C}}_{1}}{2\text{A}}\sqrt{\frac{{\text{βm}}_{\text{L}}}{{\text{V}}_{\text{c}}}}$$ (12)

Where, ω—Natural frequency of the new system, rad/s;

ξ—Damping ratio of the new system, N/(m/s).

By further establishing the mathematical model of the traditional valve-controlled system, its natural frequency and damping ratio can be obtained as:


$${\text{ω}}_{\text{t}}=\sqrt{\frac{{\text{A}}^{2}\text{β}}{{\text{V}}_{\text{c}}{\text{m}}_{\text{L}}}}$$ (13)

$${\text{ξ}}_{\text{t}}=\frac{{\text{K}}_{\text{p}}+{\text{C}}_{1}}{2\text{A}}\sqrt{\frac{{\text{βm}}_{\text{L}}}{{\text{V}}_{\text{c}}}}$$ (14)

Where, ω_t_—Natural frequency of the traditional system, rad/s;

ξ_t_—Damping ratio of the traditional system, N/(m/s).

Through the comparison of equations (11) to (14), it can be observed that the natural frequencies of the two systems are nearly identical.However, the damping ratio of the new system is mainly determined by G_p_(s). In the low-frequency range, G_p_(s) tends to 1. Consequently, the damping ratio of the new system approaches that of the traditional valve-controlled system[30].

The system is equipped with a pressure sensor that calculates the difference between the pump outlet pressure and the maximum load pressure, thereby obtaining the system’s load-sensing feedback pressure difference. The actual load-sensing pressure difference of the system is then compared to the set value of the variable-speed motor. The resulting pressure deviation is fed into a PID controller to generate an unrestricted variable-speed control signal. After speed-limiting processing, the final actual speed control value of the variable-speed motor is obtained.

The set variable-speed signal is determined by the controller, which proportionally allocates the flow to the hydraulic cylinders of different loads based on the system load conditions. The load-sensing pump adjusts the system’s required pressure based on the shuttle valve pressure difference. The variable motor adjusts its speed according to the system’s load demand. Theoretically, the variable-speed motor and the load-sensing pump can achieve stepless adjustment based on the system’s required flow and pressure. The maximum pressure can be adjusted via the relief valve. The flow to the actuator is only proportional to the flow area of the associated main valve and is independent of the load pressure[31].

## Main component analysis, calculation, and modeling

### Principle and calculation of the pressure compensator

**Fig 3:**
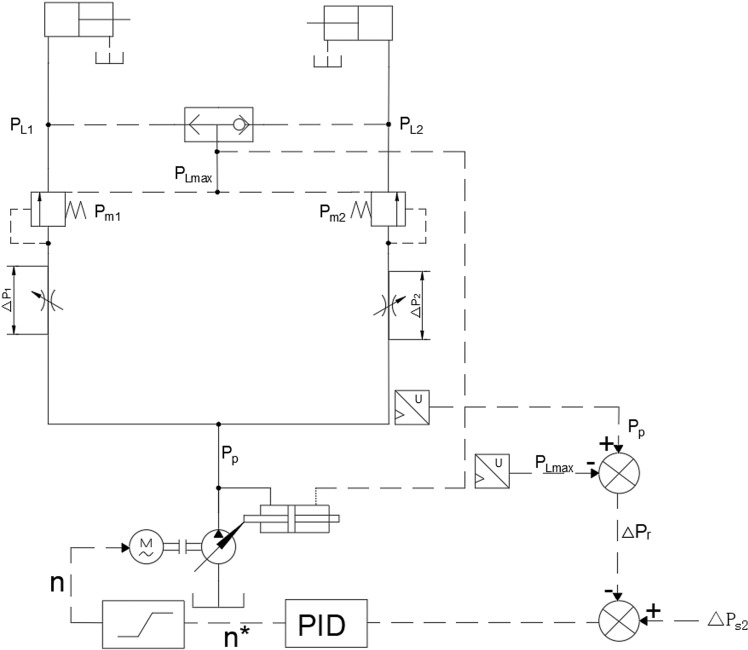
Simplified diagram of a multi-way valve principle.

To facilitate the analysis of the pressure compensation effect of the LUDV multi-way valve, a simplified LUDV system is shown in Fig 3, Where p_L1_ and p_L2_ are the load pressures, with p_L1_ being greater than p_L2_; p_m1_ and p_m2_ are the outlet oil pressures of variable throttle valves 1 and 2; _pp_ is the working pressure of the pump; and △p is the pressure difference across the variable throttle valves.One end of the pressure compensator valve spool is subjected to the outlet pressure of the variable throttle valve, while the other end is affected by the spring force and the highest load pressure. Based on the structure and force conditions of the pressure compensator, the following calculations and analysis are carried out [32].

When the pressure compensator valve spool displacement is small under low-pressure conditions, gravity, friction, and hydraulic forces can be ignored. When the pressure compensator spool is in equilibrium, the force analysis is as follows:


$$\text{p'A}-{\text{p}}_{\text{LS}}\text{A}-\text{k}\left({\text{y}}_{0}+\text{y}\right)=0$$ (15)

Where: p’ is the pressure in the p’ channel, which is the pressure after the valve;

A is the effective area of the compensating spool;

k is the spring stiffness of the pressure compensating valve;

y _0_ is the pre-compression of the compensating valve spring;

y is the displacement of the compensating spool.

Since y > 0 and y_0_ is much larger than y, according to formula (15), the pressure difference of the main spool orifice is:


$$\text{Δp}=\text{p}-\frac{{\text{p}}_{\text{LS}}\text{A}+\text{k}\left({\text{y}}_{0}+\text{y}\right)}{\text{A}}\approx \text{p}-\frac{{\text{p}}_{\text{LS}}\text{A}+{\text{ky}}_{0}}{\text{A}}$$ (16)

Since the inlet oil pressure p of each channel of the LUDV multi-way valve is equal and the load sensing pressure p_LS_ is equal, according to equation (2), the pressure difference across each throttle valve is equal. According to the flow formula, the flow rate is directly proportional to the flow area of the main valve core throttle orifice and is independent of the load pressure of each channel. The flow rate of the multi-way valve can be described by the Bernoulli flow equation for flow through the throttle orifice:


$${\text{Q}}_{\text{i}}={\text{C}}_{\text{d}}\cdot {\text{A}}_{\text{i}}\cdot {\text{Δp}}_{\text{i}}^{\text{k}}$$ (17)

Where: Q_i_ is the flow rate through the i-th section;

C _d_ is the flow coefficient, determined by the valve design;

A _i_ is the effective area of the valve port, which depends on the spool position;

k is a coefficient.

Thus, in a load-sensing system, the operating speed of the actuator is not affected by the load size but is determined by the valve port opening of the directional valve. As the valve port opening increases, the actuator speed increases accordingly. Applying this system to agricultural machinery can effectively improve its performance.For the load-sensing functionality achieved by the multi-way valve, the system can provide feedback on demand and adjust the hydraulic pump. The pump adjusts the provided flow and pressure according to the load demand, thereby reducing energy consumption [33].

### Establishment and simulation of the compensator model

The pressure compensator mainly consists of three parts: the valve body, valve spool, and spring.The spring force is set as a fixed value, and the pressure difference across the main control valve (upstream and downstream of the valve) remains constant. The flow rate to the flow control valve is only related to the spool opening of the control valve. The essence of upstream pressure compensation is essentially a speed regulation circuit based on a constant-pressure-reduction valve [34].

The motion differential equation of the pressure compensating valve is:


$$\left({\text{p}}_{\text{s}1}-{\text{p}}_{\text{L}}\right){\text{A}}_{3}-{\text{K}}_{\text{c}}{\text{x}}_{2}-{\text{F}}_{1}={\text{M}}_{1}\frac{{\text{d}}^{2}{\text{x}}_{2}}{\text{dt}}$$ (18)

In the equation, ps1 represents the outlet pressure of the pressure compensating valve, in Pa;

P_L_ is the pressure fed back to the pressure compensating valve by the load, in Pa;

K_c_ is the spring stiffness coefficient of the pressure compensating valve, in N/mm;

x_2_ is the spool displacement function of the pressure compensating valve, in mm;

M_1_ is the mass of the pressure compensating valve, in kg;

A_3_ is the area of the spool of the pressure compensating valve, in mm^2^;

F_1_ is the spring preload of the pressure compensating valve, in N.

Applying the Laplace transform to the above equation yields:


$$\left({\text{P}}_{\text{S}1}-{\text{P}}_{\text{L}}\right){\text{A}}_{3}-{\text{K}}_{\text{c}}{\text{X}}_{2}={\text{M}}_{1}{\text{s}}^{2}{\text{X}}_{2}$$ (19)

The flow continuity equation for a pressure compensating valve is:


$${\text{Q}}_{\text{L}}={\text{K}}_{\text{lq}}{\text{X}}_{2}-{\text{K}}_{1\text{p}}{\text{P}}_{\text{Sl}}$$ (20)

In the formula, K_1q_ is the flow gain coefficient of the pressure compensating valve;

K _1p_ is the flow-pressure gain coefficient of the pressure compensating valve;

Q _L_ is the outlet flow of the pressure compensating valve, L/min;

P_s1_ is the outlet pressure of the pressure compensating valve, Pa.

**Fig 4:**
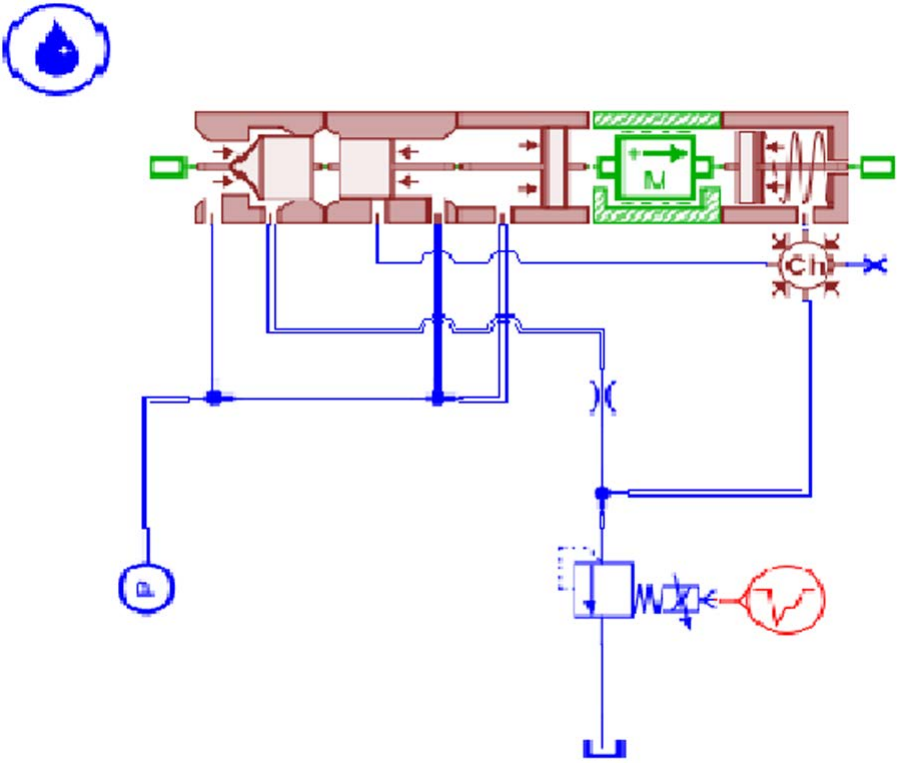
Pressure Compensator Model.

The simulation model of the pressure compensator is shown in Fig 4. The inlet of the pressure compensator is connected to a constant pressure source, with the constant pressure set to 10 MPa. A relief valve is used to simulate the variation in load pressure, with the relief valve pressure set to increase from 0 MPa to 10 MPa within 10 seconds. The precompression force of the pressure compensator spring is set to 100 N. The simulation results show the pressure changes upstream and downstream of the throttle valve. The main parameters of the pressure compensator are shown in Table 1.

**Table 1:** Main Parameters of the Pressure Compensator.

Number sequence	Parameter name	Parameter value
1	Spool mass/(kg)	0.01
2	poppet half angle/(degree)	15
3	spool diameter/(mm)	19.5
4	rod diameter/(mm)	5
5	underlap corresponding to zero displacement/(mm)	2
6	hole diameter/(mm)	5
7	spring force at zero displacement/(N)	100

**Fig 5:**
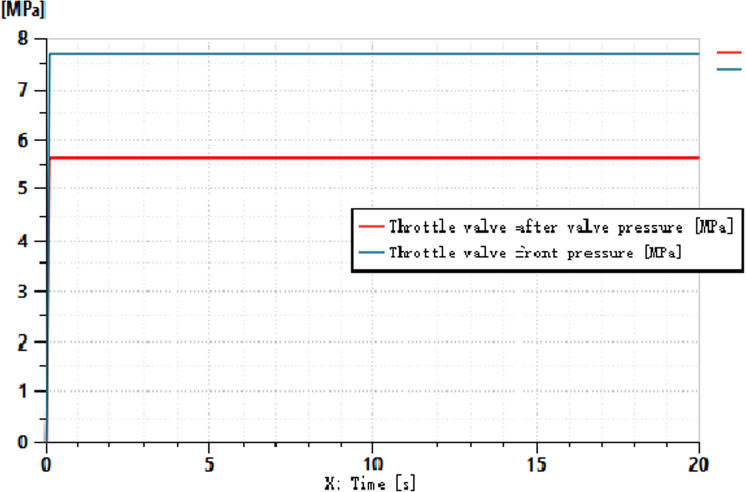
Pressure Variation Before and After the Throttle Valve.

As shown in Fig 5, with the variation in load pressure, the pressure upstream of the throttle valve increases accordingly, while the pressure difference across the throttle valve remains approximately constant at around 2.2 MPa. At this point, the flow rate through the throttle valve remains stable.

### Establishment and Simulation of the Load-Sensing Pump Model

The load-sensing system enables the hydraulic pump to sense load variations and provide a corresponding flow rate in a timely manner. This system offers advantages such as high operational efficiency and low power loss. During the operation of actuators in a load-sensing system, the operating speed of the actuators depends only on the valve port opening of the directional valve and does not vary with load changes. Therefore, by controlling the throttle opening, the load-sensing system achieves excellent energy-saving performance. The hydraulic pump can continuously maintain the flow required by the load, enabling the control of multiple actuators without mutual interference during operation. Additionally, the control system exhibits fast response speed and high precision [35].

**Fig 6:**
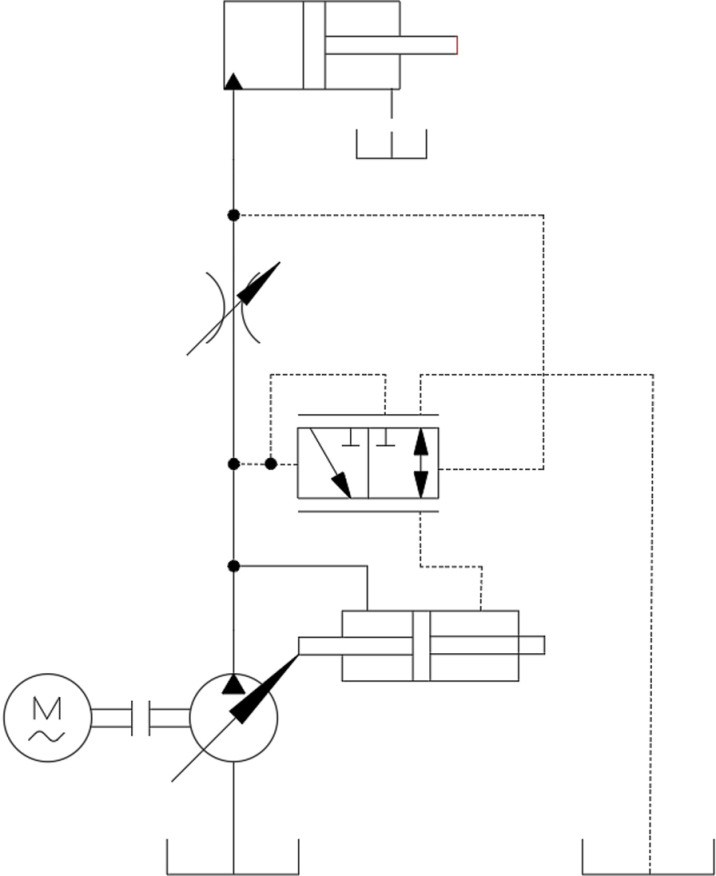
Schematic diagram of load-sensitive pump.

In the load-sensing system, the load pressure is directly introduced into the load-sensing valve. The load pressure maintains a certain value relative to the system input pressure. Based on load variations, the output pressure of the variable pump is adjusted in real-time. The flow output from the directional valve depends only on the main valve flow area. The principle of the load-sensing pump is illustrated in Fig 6.

**Fig 7:**
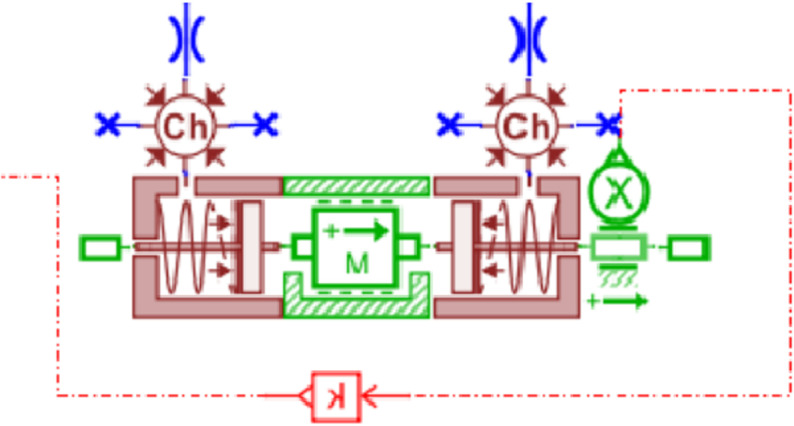
Differential Pressure Displacement Detection Device.

In the initial state of the system, hydraulic oil flows through the throttle port into the left chamber of the load-sensing valve. Under the action of hydraulic pressure, the valve spool moves to the right, overcoming the spring force in the right spring chamber. The load-sensing pump receives the LS pressure from the oil circuit of the directional valve, which passes through the variable throttle port and enters the right chamber of the load-sensing valve. This continues until the pressures on the left and right sides of the valve spool reach equilibrium. At this point, the main oil circuit of the load-sensing valve is connected, and the load-sensing pump, equipped with a pressure difference displacement detection device, operates as illustrated in Fig 7.

**Fig 8:**
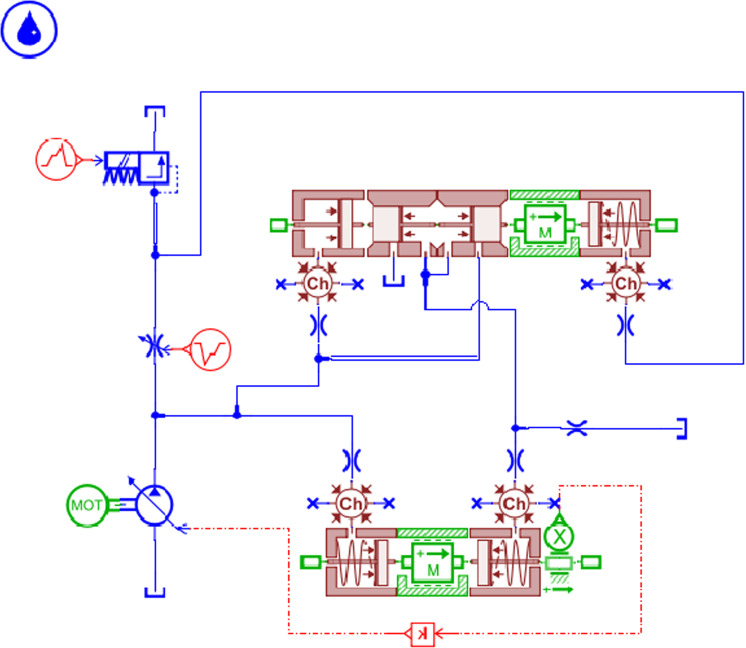
Load-Sensitive Pump Model.

The pressure differential-displacement detection device mainly consists of a force comparison piston, a compression spring, and a displacement sensor.The left spring chamber is a rod chamber, the right spring chamber is a rodless chamber, and the piston diameter is 20mm, with key parameters listed in Table 2. The pressure in the spring chamber on the left side of the detection device is derived from the variable pump outlet pressure after passing through the throttle orifice, while the pressure in the spring chamber on the right side is sourced from the main oil circuit pressure of the load-sensing valve. The hydraulic force in the right spring chamber is influenced by the LS pressure; as the LS pressure increases, the opening of the load-sensing valve decreases, leading to a reduction in pressure in the right spring chamber. Conversely, a decrease in LS pressure results in an increase in the pressure within the right spring chamber. The AMESim model of the load-sensing pump is illustrated in Fig 8.

**Table 2:** Parameters of Differential Pressure Displacement Detection Device.

Number sequence	Parameter name	Parameter value
1	Left chamber rod diameter/(mm)	15
2	Left chamber zero displacement spring compression/(mm)	10
3	spring force at zero displacement/N	20
4	spring rate/(N/mm)	20
5	Spool mass/(kg)	0.1
6	coefficient of viscous friction/(N·s/m)	80
7	Left chamber rod diameter/(mm)	15

**Fig 9:**
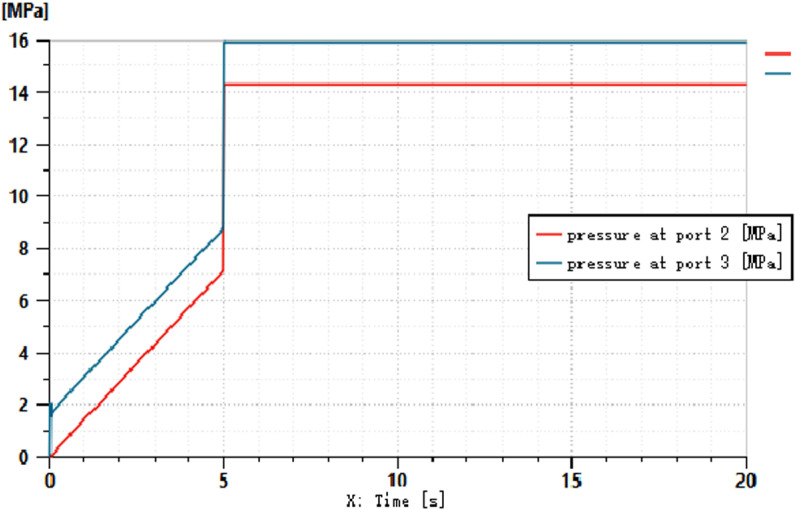
Pressure before and after the variable throttle valve.

**Fig 10:**
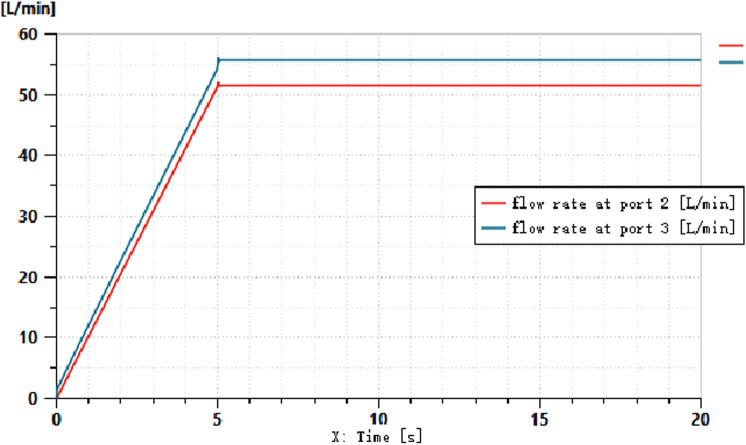
Flow Curve Before and After the Variable Throttle Valve.

The simulation conditions are set as follows: the speed of the driving motor is set to 1500 r/min, the displacement of the load-sensitive pump is 100 ml/r, the pressure of the relief valve is set to 25 MPa, and the maximum diameter of the variable throttle orifice is 5 mm. During the signal input from 0 to 5 seconds, the variable throttle valve gradually opens. Through the simulation, the pressure and flow curves of the load-sensitive pump can be obtained, as shown in Figs 9 and 10.

As shown in Figs 9 and 10, when a variable signal is applied to the throttle orifice, the load-sensitive pump dynamically balances the hydraulic force through the valve spool, directing it to the right chamber of the differential displacement device. This action allows the differential displacement detection device to output a signal that controls the output pressure of the variable pump. With the change in the throttle opening, the pressure before and after the throttle valve increases accordingly. At the fifth second, the load doubles, but the throttle opening does not increase further. From this, it can be inferred that the pressure differential across the throttle valve remains approximately at 2 MPa, while the flow rate through the throttle valve remains constant.

## Establishment and analysis of the dual-variable load independent flow distribution multiway valve system model

### Establishment and analysis of the load independent flow distribution multiway valve system

**Fig 11:**
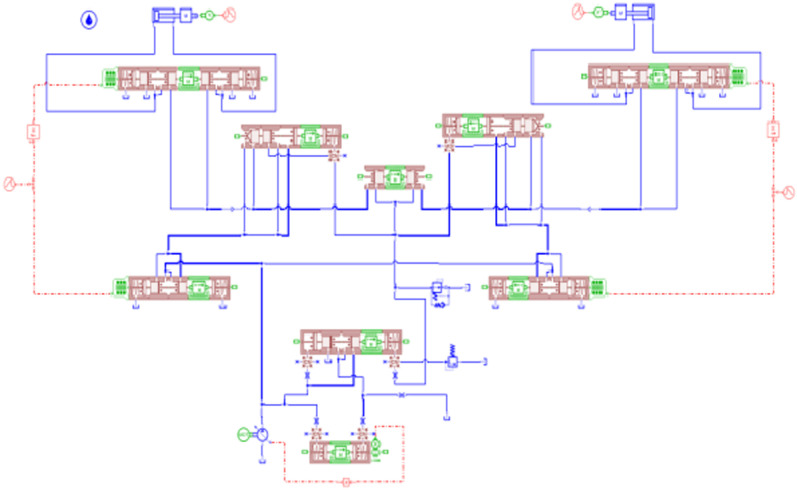
Hydraulic System Simulation Model.

**Fig 12:**
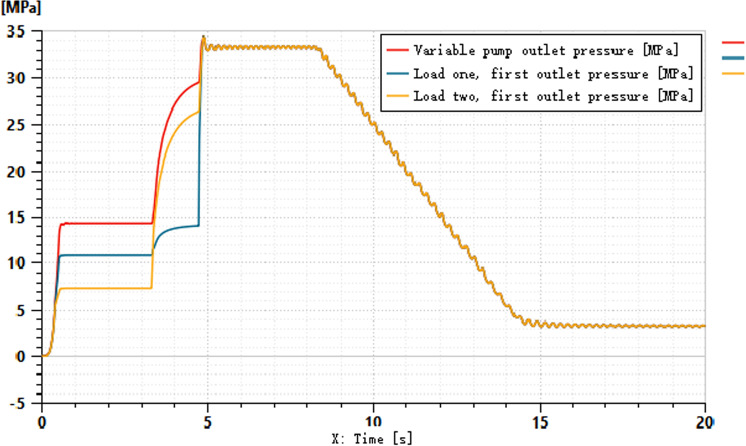
Hydraulic System Pressure.

**Fig 13:**
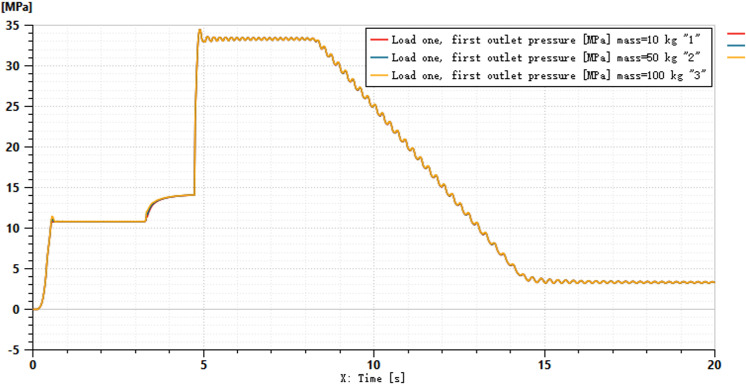
Pressure in hydraulic cylinder under load.

**Fig 14:**
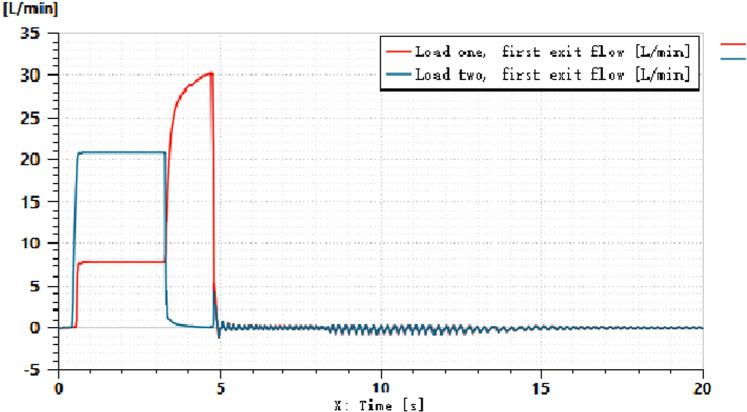
Hydraulic Cylinder Flow.

A simulation model of the load-independent flow distribution multiway valve system is established, as shown in Fig 11. The hydraulic cylinder load is simulated by applying an additional pressure signal to represent the soil resistance load encountered during actual tillage operations, with M denoting the total mass of the suspended rod and agricultural implements. The motor speed is set to 1500 r/min, and the simulation time is 20 seconds. The input friction force for the left hydraulic cylinder is set to 5000 N, while the right side is set to 2500 N, with a control signal applied for 5 seconds. The input signal for the direction control valve is 10 mA, and for the main directional valve, it is 100 mA;For ease of calculation, assume that the mass of all spool is 0.01 kg.the inner diameter of the main directional valve is 15 mm, and the rod diameter is 5 mm. The relationship between the variable pump outlet and the pressure changes across the two loads is illustrated in Fig 12. Different load masses of 10 kg, 50 kg, and 100 kg are set, resulting in the hydraulic cylinder piston pressure curves under varying external loads, as shown in Fig 13, and the hydraulic cylinder flow rate curves, as depicted in Fig 14.

As shown in Fig 12, at the initial moment, when the directional valve opens, the pump outlet pressure rises sharply until approximately 0.59 seconds, when it begins to stabilize. At around 3.08 seconds, the pressure starts to rise again until it stabilizes once more around the 5-second mark. Under the influence of the load-sensitive pump and the pressure compensator, the pressure begins to decrease uniformly at 8 seconds, eventually stabilizing at approximately 3.4 MPa.

Fig 13 illustrates the load pressure conditions under different resistances. When combined with the pressure situation depicted in Fig 12, it can be observed that the variable pump outlet pressure exceeds the pressures across both loads. Due to the action of the pressure compensator, the variable pump adjusts its input pressures differently for the two distinct loads.When a variable load pressure is applied to one of the hydraulic cylinders, it is evident that the hydraulic cylinder does not exhibit changes in load pressure due to differing forces. The load pressure curves demonstrate good engagement, resulting in relatively stable pressure profiles.

Fig 14 indicates that the system effectively allocates flow rates to the hydraulic cylinder under different pressure loads. Coupled with the pressure curve from Fig 12, it is evident that the system dynamically adjusts the output pressure and real-time flow distribution.

**Fig 15:**
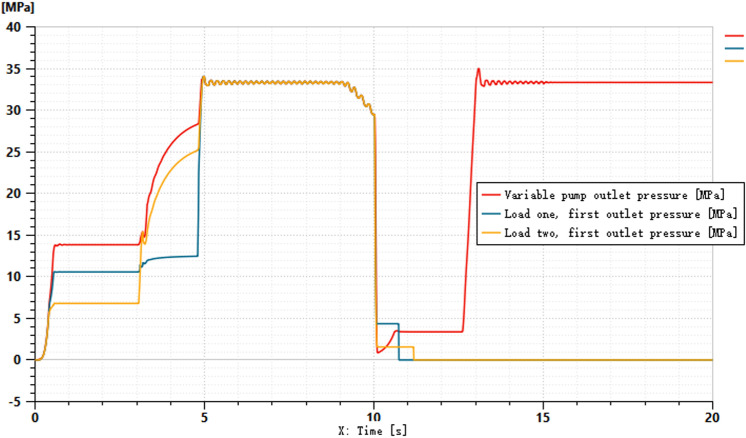
Pressure Variation at Port 1.

**Fig 16:**
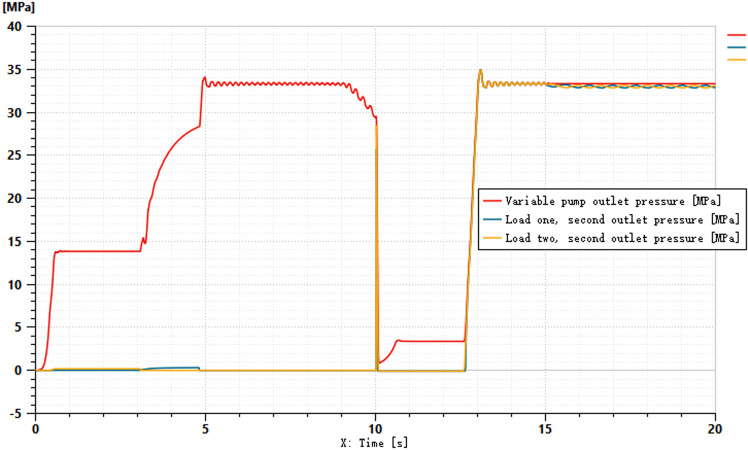
Pressure Variation at Two Ports.

To better accommodate the practical application of tractors in hilly and mountainous areas, the simulation incorporates the scenarios of spool shifting and system idling. Other system parameters remain unchanged, and the simulation duration is set to 20 seconds. Within the time interval of 1–10 seconds, the control signal for the main directional valve is set to 100 mA. Between 10–15 seconds, the control signal is adjusted to -100 mA, and from 15–20 seconds, the control signal is set to 0 mA. The purpose of applying positive and negative control currents is to simulate the lifting and lowering states of the suspension system. The control signal of 0 mA represents the completion of the operation, where the system remains in a suspended state. For the hydraulic cylinder, port 1 is designated as the inlet for lifting, while port 2 serves as the inlet for lowering. The pressure variations at ports 1 and 2, as well as the outlet pressure of the variable pump, are shown in Figs 15 and 16.

Fig 15 shows the pressure curve at port 1 of the two hydraulic cylinders. From the curve, it can be observed that when the input signal is 100 mA, the pressure remains constant during the first 10 seconds. After 10 seconds, the pressure does not drop directly to zero but is reduced in stages to mitigate excessive impact. Around 11 seconds, the pressure at port 1 for both loads drops to zero. Fig 16 illustrates the return pressure variation at port 2 of the two hydraulic cylinders. When the spool shifts at 10 seconds, there is a slight fluctuation in pressure. Around 12.5 seconds, the pressure at port 2 of both hydraulic cylinders increases. Between 15–20 seconds, the main spool remains in the neutral position, during which the pressure at port 1 of both cylinders drops to 0 MPa, while the pressure at port 2 stabilizes at approximately 33 MPa.

### Establishment and analysis of dual-variable controlled load sensing system

**Fig 17:**
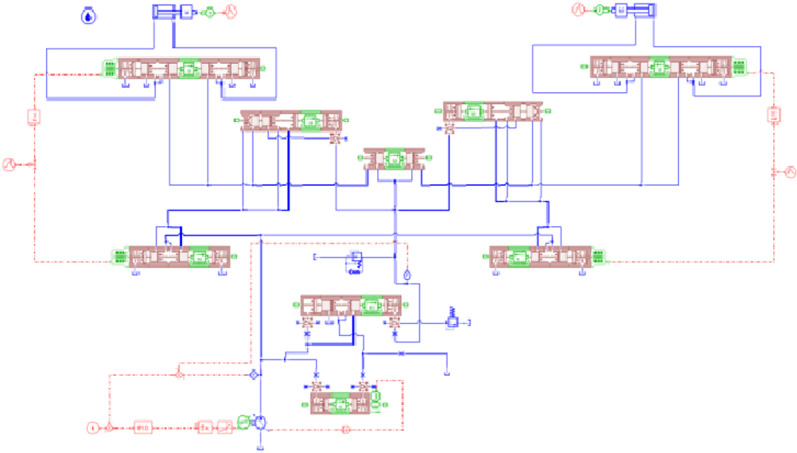
Dual-variable controlled load sensing system.

Dual-variable controlled load sensing system is established as shown in Fig 17. This system builds upon the load-independent flow distribution multi-way valve system by incorporating variable motor control, forming Dual-variable controlled load system in conjunction with the load-sensitive pump.The pressure sensor calculates the required displacement of the system, determining the system’s pressure deviation. This deviation is then sent to the PID controller to generate a variable speed signal. By processing this signal, the actual rotational speed of the variable motor can be obtained.

**Fig 18:**
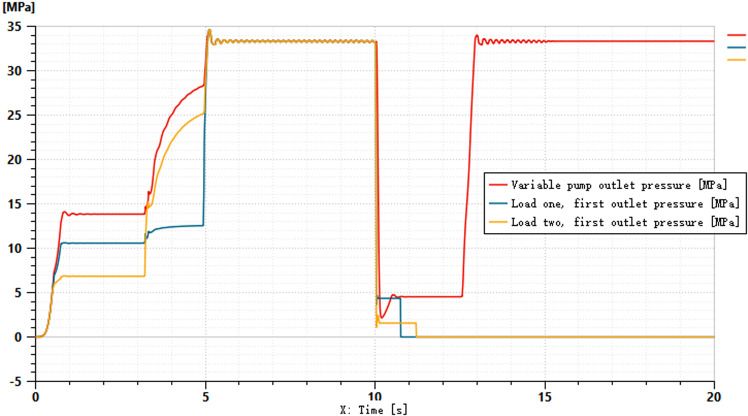
Pressure at Oil Port 1 of the Two-Variable System Load.

**Fig 19:**
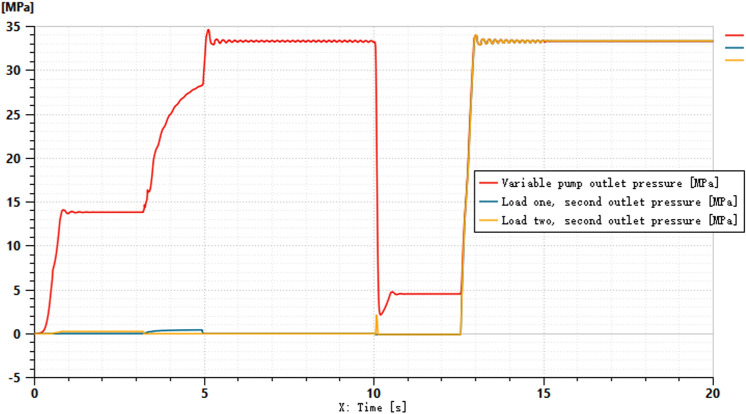
Pressure at the oil port 2 of the two-variable system load.

**Fig 20:**
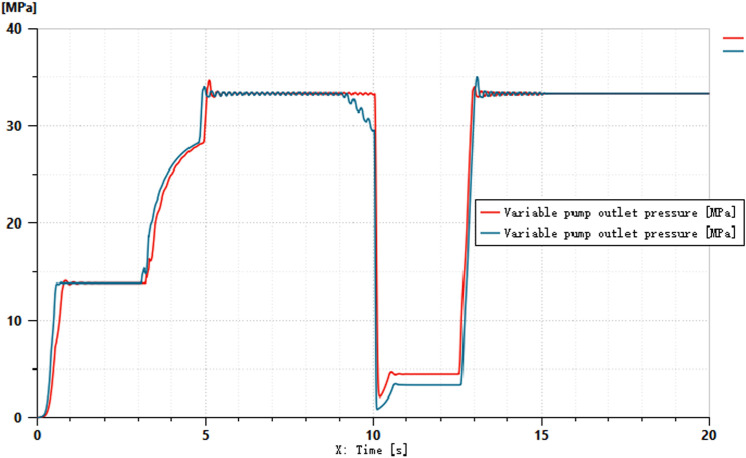
Variable Pump Outlet Pressure.

**Fig 21:**
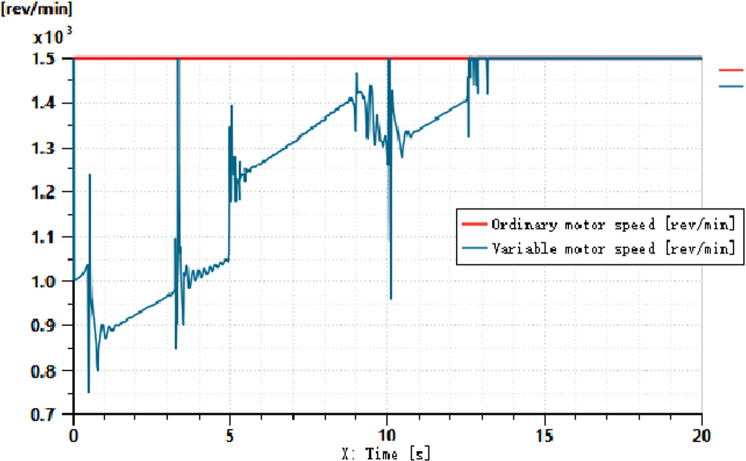
Motor Speed Comparison.

The pressures at ports 1 and 2 of the dual-variable system load are shown in Figs 18 and 19, respectively. From the figs, it can be observed that the pressure trends at ports 1 and 2 in the dual-variable system are nearly identical to those in the single-variable system. Taking the pump outlet pressure as an example, the comparison between the dual-variable system pump outlet pressure and the single-variable system pump outlet pressure is shown in Fig 20. The dual-variable system shows a slightly delayed startup compared to the single-variable system, but the response delay is less than 0.1 seconds. At 10 seconds, when the directional change occurs, the dual-variable system responds slightly faster than the single-variable system, with no significant impact on the overall pressure change trend for either system. Prior to the spool shifting, the pressure in the single-load system decreases slightly due to the flow distribution within the system. In terms of load stability, the dual-variable system outperforms the single-variable system.Overall, although the dual-variable system exhibits a slightly slower response time, it demonstrates superior load stability compared to the single-variable system. The comparison of the actual real-time speed of the variable-speed motor versus the regular motor during the simulation is shown in Fig 21.

**Fig 22:**
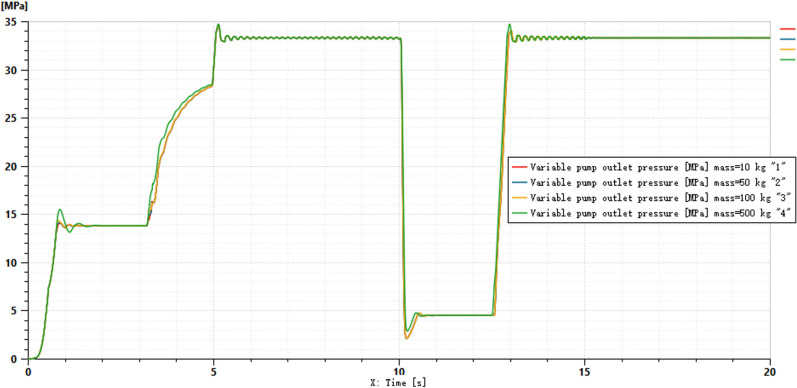
Variable Load Pressure Variable Pump Output Pressure.

From the motor speed comparison shown in Fig 21, combined with Figs 20 and 21, it can be concluded that under the condition of changing motor speed, the outlet pressure of the variable pump shows no significant variation. However, the energy efficiency of the variable-speed motor demonstrates considerable advantages, achieving an average energy savings of up to 80% compared to the standard fixed-speed motor, with a maximum savings of up to 50%. The outlet pressure of the variable pump under different load conditions is illustrated in Fig 22.

As shown in Fig 22, the graph illustrates the variation in pump outlet pressure under four different mass conditions. During the initial phase (0 to approximately 5 seconds), the pressure of all curves rises rapidly, although the rate of increase and the final stabilized pressure values differ. Over time, the pressure gradually stabilizes, with all curves eventually reaching the same pressure level regardless of the mass differences. Under varying load pressures, the variable pump output pressure in the dual-variable load sensing system remains consistent. Therefore, the output pressure of the dual-variable system does not change with load variations, with fluctuations maintained within the designed 5% error margin of the target pressure.

## Conclusion

Focusing on the LUDV hydraulic system of hilly and mountainous tractors, this study proposes a dual-variable control load-sensing system and draws the following conclusions:

1. A physical simulation model of the dual-variable control load-sensing system, incorporating a variable pump and a variable-speed motor, was developed using AMESim software. The accuracy of the model was validated, demonstrating the independent flow distribution characteristics of the multi-way valve. The system can proportionally distribute flow to each actuator according to the operational signals under varying load conditions without mutual interference among the actuators. This effectively enhances the system’s control precision and response speed.
2. Compared to a single-variable pump load-sensing system, the designed dual-variable control load-sensing system exhibits superior energy-saving performance, achieving an average energy-saving effect of 20% and a maximum savings of up to 50%. This improvement is attributed to the system’s ability to adjust the variable pump’s output flow and motor speed in real time based on pressure difference and displacement signals, enabling adaptive matching to the pressure requirements of the load system.
3. The dual-variable system demonstrates significant advantages in maintaining operational pressure stability and flow control accuracy. It effectively limits pressure fluctuations to within a 5% error margin of the design pressure, ensuring reasonable flow distribution for hydraulic cylinders under different load conditions. Additionally, the system dynamically adjusts output pressure in real time, meeting the operational requirements of hilly and mountainous tractors when working in complex terrains.

## Supporting information

S1 Data Data for parameters. (XLSX)
